# GDF11 expressed in the adult brain negatively regulates hippocampal neurogenesis

**DOI:** 10.1186/s13041-021-00845-z

**Published:** 2021-09-06

**Authors:** Brittany A. Mayweather, Sean M. Buchanan, Lee L. Rubin

**Affiliations:** 1 Department of Stem Cell and Regenerative Biology, Harvard University, Cambridge, MA USA; 2 Graduate Program in Biological and Biomedical Sciences, Harvard Medical School, Boston, MA USA; 3 Harvard Stem Cell Institute, Sherman Fairchild Bldg, 7 Divinity Ave., Cambridge, MA 02138 USA

**Keywords:** GDF11, Adult hippocampal neurogenesis, Neural progenitor cells, Proliferation, Knockout mouse

## Abstract

Growth differentiation factor 11 (GDF11) is a transforming factor-β superfamily member that functions as a negative regulator of neurogenesis during embryonic development. However, when recombinant GDF11 (rGDF11) is administered systemically in aged mice, it promotes neurogenesis, the opposite of its role during development. The goal of the present study was to reconcile this apparent discrepancy by performing the first detailed investigation into the expression of endogenous GDF11 in the adult brain and its effects on neurogenesis. Using quantitative histological analysis, we observed that *Gdf11* is most highly expressed in adult neurogenic niches and non-neurogenic regions within the hippocampus, choroid plexus, thalamus, habenula, and cerebellum. To investigate the role of endogenous GDF11 during adult hippocampal neurogenesis, we generated a tamoxifen inducible mouse that allowed us to reduce GDF11 levels. Depletion of *Gdf11* during adulthood increased proliferation of neural progenitors and decreased the number of newborn neurons in the hippocampus, suggesting that endogenous GDF11 remains a negative regulator of hippocampal neurogenesis in adult mice. These findings further support the idea that circulating systemic GDF11 and endogenously expressed GDF11 in the adult brain have different target cells or mechanisms of action. Our data describe a role for GDF11-dependent signaling in adult neurogenesis that has implications for how GDF11 may be used to treat CNS disease.

**Supplementary Information:**

The online version contains supplementary material available at 10.1186/s13041-021-00845-z.

## Background

GDF11 is a member of the transforming growth factor-β (TGF-β) superfamily of cytokines and acts as a critical regulator of neurogenesis during central nervous system (CNS) development [1, 2]. During embryogenesis, GDF11 largely functions as a negative growth regulator through its inhibition of neural progenitor cell (NPC) proliferation and promotion of neuronal differentiation. For example, in the embryonic olfactory epithelium, GDF11 promotes differentiation into mature olfactory receptor neurons by inducing cell cycle arrest of proliferative NPCs [3–5]. In the developing retina, GDF11 again regulates numbers of neurons, but by controlling the period of differentiation of progenitors, rather than their proliferation [6]. And in the developing spinal cord, GDF11 expression by newborn neurons triggers the cell cycle exit of adjacent NPCs which facilitates their crucial transition from producing early born neurons to late born neurons and oligodendrocytes [7].

Recent studies utilizing recombinant GDF11 (rGDF11) suggest GDF11 signaling may continue to influence neurogenesis well beyond embryogenesis. Surprisingly, systemic administration of rGDF11 into aged mice was shown to promote, rather than inhibit, neurogenesis within both adult neurogenic niches [8, 9]. While the therapeutic implications of rGDF11 administration are exciting, questions have been raised about how to reconcile the pro-neurogenesis activities of systemically administered rGDF11 with the anti-neurogenesis functions of embryonic brain derived GDF11. One possibility is that, in the adult, GDF11 crosses the blood–brain barrier and stimulates, rather than inhibits, neurogenesis. The second possibility is that systemic GDF11 does not enter the brain but exerts a pro-neurogenic effect in another way, possibly by acting on vascular cells which are known to secrete neurogenesis-regulating factors. This latter mechanism is consistent with our observation that GDF11 has, at best, a limited ability to cross the BBB.

To help resolve this issue, we investigated GDF11 expression and function in the adult brain, which had not been examined in much detail. We first performed a detailed histological analysis of *Gdf11* expression in many regions of the adult mouse brain. Using adult transgenic mice deficient in *Gdf11*, we then investigated the role of endogenous GDF11 during adult hippocampal neurogenesis. In the absence of *Gdf11*, we observed a dramatic increase in proliferation and an increased number of neural progenitor cells in the hippocampus, but significantly fewer newborn neurons. Our results suggest that GDF11 expressed in the adult brain acts as a negative regulator of neurogenesis, as it does during development. By demonstrating that endogenous GDF11 in the CNS inhibits adult NPC proliferation, we provide further support for the hypothesis that systemically administered rGDF11 promotes neurogenesis indirectly, in line with our previous data showing GDF11 does not cross the BBB.

## Results

### *Gdf11* expression in neurogenic and non-neurogenic regions of the adult brain

**Fig. 1:**
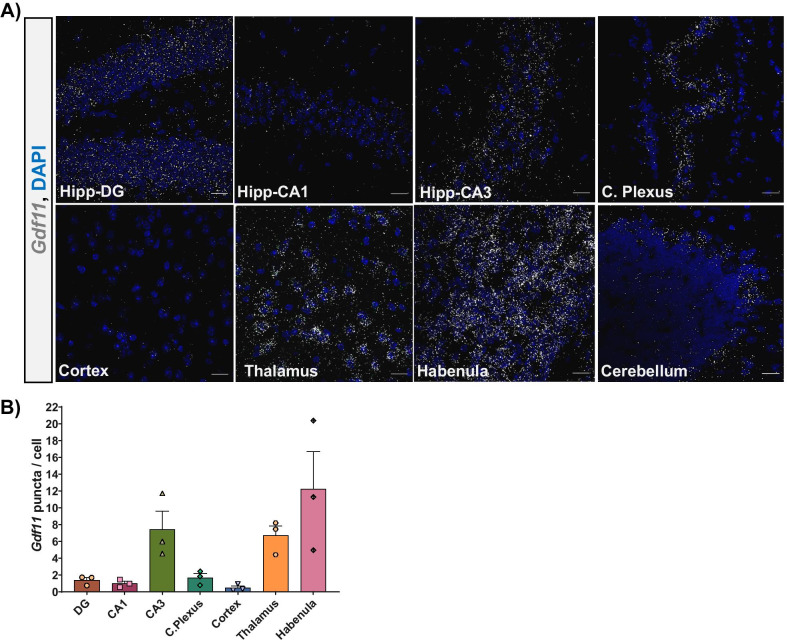
*Gdf11* is expressed throughout the young adult brain. **A** Representative RNAscope micrographs from a 3-month-old male C57BL/6 mouse probed for *Gdf11* (white puncta) and stained with DAPI (blue) for the following brain regions: hippocampus dentate gyrus (DG), CA1, CA3, cortex, thalamus, habenula, and cerebellum. Scale bar = 20 μm. **B** Bar graph depicting the quantification of RNAscope data (mean of 3–4 mice per brain region, error bars represent SEM)

Previous studies which profiled the CNS expression of *Gdf11* largely focused on embryonic and early postnatal brains [1, 2]. To examine the localization of *Gdf11* in the mature brain, we prepared sections from 3-month-old young adult mice and performed RNAscope in-situ hybridization with a probe against *Gdf11*. Overall, *Gdf11* was highly expressed in the hippocampus, choroid plexus, thalamus, habenula, and cerebellum, relative to its much lower expression in the cortex (Fig. 1A). We quantified *Gdf11* expression across regions using CellProfiler and observed fourfold and eightfold more *Gdf11* in the choroid plexus and hippocampus, respectively compared to the cortex (Fig. 1B). We observed 22-fold more *Gdf11* in the thalamus compared to the cortex. The habenula subregion represented the brain area with the highest density of *Gdf11* (Fig. 1B). Within the cerebellum, *Gdf11* selectively localized along the Purkinje cell layer while being largely absent from the molecular layer or fiber tracts (Fig. 1A).

**Fig. 2:**
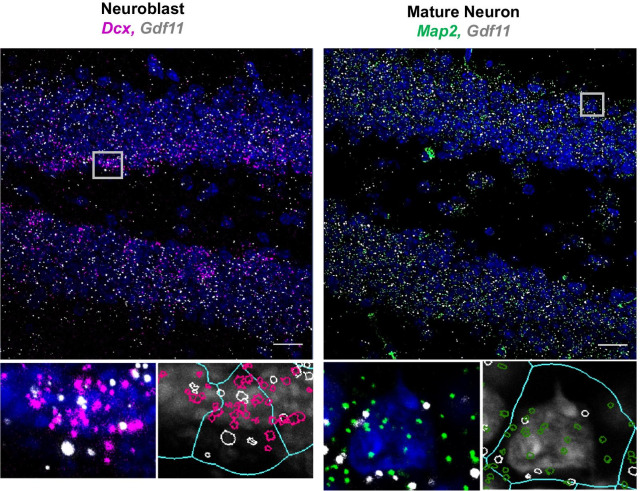
*Gdf11* is expressed by neuroblasts and mature neurons in the adult dentate gyrus. Representative RNAscope micrographs from a 3-month-old male C57BL/6 mouse probed for *Gdf11* (white puncta) cell type specific markers for neuroblasts (*Dcx*, *pink*) or mature neurons (*Map2*, *green*) and stained with DAPI (blue). Gray boxes mark the regions chosen for the inserts below each micrograph shown. Scale bar = 20 μm. Inserts are magnified DAPI stained nuclei (grey) with each puncta outlined by either white, pink, or green. A light blue overlay outlines the perinuclear area used to determine puncta coexpression

Given the important role of GDF11 in regulating embryonic neurogenesis, we were particularly interested in profiling zones of the brain where neurogenesis continues into adulthood. This includes the subventricular zone (SVZ) and the subgranular zone (SGZ) within the hippocampus. We observed consistently high levels of *Gdf11* within the lateral ventricle choroid plexus and the adjacent SVZ (Fig. 1A). Co-expression data with cell type specific markers showed that neuroblasts and choroid plexus epithelial cells, but not endothelial cells, are likely the primary sources of *Gdf11* in the choroid plexus region (Additional file 1: Fig. 1A). In the hippocampus, *Gdf11* was expressed in the CA1, CA3, and dentate gyrus (DG), with the highest expression found in the CA3 pyramidal cell bodies (Fig. 1A). Of note is *Gdf11*’s relatively uniform distribution across the DG, with expression observed within granule cell bodies and within the neurogenic SGZ. To determine if *Gdf11* is expressed in neuronal progenitor cells and/or mature neurons in the dentate gyrus, we performed RNAscope co-expression studies. We combined a probe against *Gdf11* with probes which label neuroblasts (*Dcx*) or mature neurons (*Map2*) (Fig. 2)**.** We found that the majority of neuroblasts and neurons in the dentate gyrus were positive for *Gdf11.* In addition, we explored *Gdf11* expression in non-neuronal cells. *Gdf11* was present in oligodendrocyte progenitor cells (Pdgfra+, Olig1+), but largely absent from mature oligodendrocytes (Pdgfra−, Olig1+). Few, if any, *Gdf11* puncta were detected within nuclei identified as microglia or endothelial cells (Additional file 1: Fig. 1B). GDF11 is highly homologous at the amino acid level with MSTN (GDF8), and there has been some controversy regarding functional differences between the two proteins in peripheral tissues. However, within the dentate gyrus, *Mstn* was expressed at substantially lower levels than *Gdf11* (Additional file 1: Fig. 2), and we did not pursue its actions further.

Since hippocampal neurogenesis declines significantly with age, we were interested in whether *Gdf11* expression there also changes over time [10]. We prepared sections from 18 month old mice, performed RNAscope, quantified *Gdf11* expression in the hippocampal DG, CA1, and CA3 and compared these results with those obtained from 3 month old mice (Additional file 1: Fig. 3A). Surprisingly, we observed no statistically significant change in *Gdf11* expression in any hippocampal region studied (Additional file 1: Fig. 3B). We also performed qPCR on isolated whole hippocampal tissue from young and old mice and again observed no change in *Gdf11* expression (Additional file 1: Fig. 3C). These findings suggest that age-associated changes in hippocampal neurogenesis are not a direct result of changes in the hippocampal expression of *Gdf11* itself.

### Construction and characterization of *Gdf11*^*cKO*^ mice

The expression of *Gdf11* in neurons, neuroblasts and the dentate gyrus led us to hypothesize that it may continue to play a role in hippocampal neurogenesis even in adult mice. To investigate this, we generated tamoxifen inducible GDF11 knockout mice (*Gdf11*^*cKO*^), which avoid the perinatal lethality that occurs in germline *Gdf11*^*−/−*^ mice [1, 2]. Mice homozygous for *loxP*-flanked GDF11 (*Gdf11*^flox/flox^) were crossed with transgenic mice expressing a tamoxifen inducible recombinase (Cre-ER) using the chicken beta actin promoter/enhancer coupled with the cytomegalovirus immediate-early enhancer (CAGGCre-ER™) [1, 11]. A ubiquitously expressed Cre-ER driver was chosen to ensure widespread depletion of *Gdf11* across the brain, accounting for both neuronal and non-neuronal cellular sources of GDF11. All experiments described below involved 3-month-old male and female mice injected with tamoxifen once daily for three consecutive days and sacrificed 6 weeks later.

**Fig. 3:**
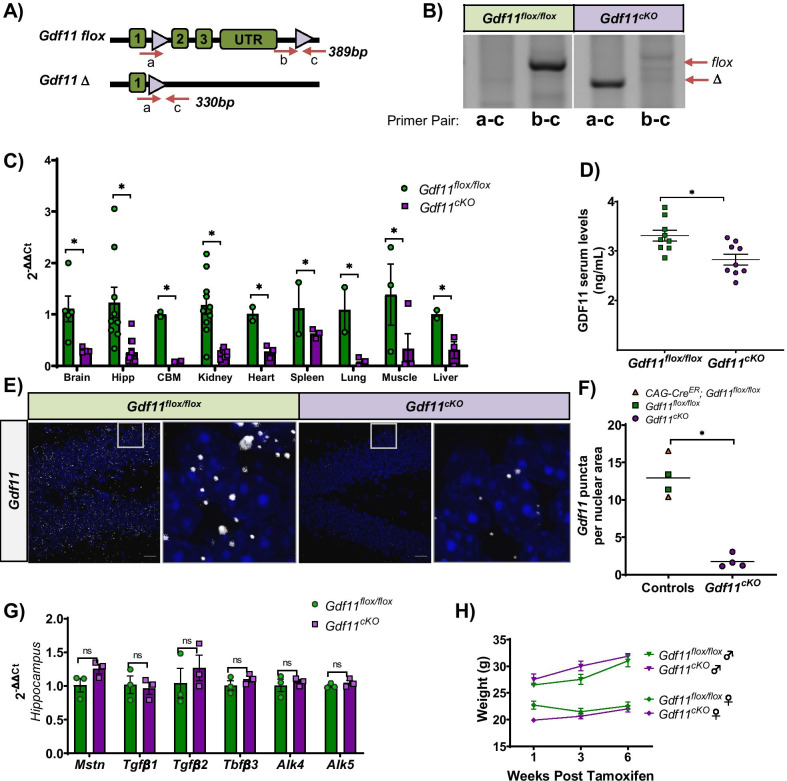
Construction and characterization of *Gdf11*^*cKO*^ mice. **A** Schematic of *Gdf11 flox* (top), and *Gdf11* ∆ (bottom) alleles. Primer binding sites are labeled by a, b, and c alongside their amplicon sizes. **B** Genomic DNA PCR for *Gdf11 flox* (b-c amplicon: 389 bp) and *Gdf11* ∆ (a-c amplicon: 330 bp) amplicons from representative *Gdf11*^*flox/flox*^ and *Gdf11*^*cKO*^ mice. These data confirm recombination of the *Gdf11* floxed allele in the *Gdf11*^*cKO*^, but not *Gdf11*^*flox/flox*^, mice. **C** Quantitative PCR analysis of *Gdf11* expression in whole brain, hippocampus, cerebellum, kidney, heart, spleen, lung, muscle (tibialis anterior), and liver tissue isolated from *Gdf11*^*flox/flox*^ and *Gdf11*^*cKO*^ mice. *Hprt* was used as a housekeeping gene. Relative mRNA levels for all samples are normalized to *Gdf11*^*flox/flox*^ mice and error bars depict SEM. **D** Concentration of GDF11 protein in serum of *Gdf11*^*flox/flox*^ and *Gdf11*^*cKO*^ mice at 3 months old, as detected by mass spectrometry (n = 9 mice per genotype, males and females pooled.) Individual data points are overlaid with mean ± SEM. **E** RNAscope in situ hybridization micrographs of mouse hippocampi (dentate gyrus) probed for *Gdf11* (white puncta) from representative *Gdf11*^*flox/flox*^ and *Gdf11*^*cKO*^ mice. Scale bar = 20 μm. **F** Scatterplot showing the quantification of RNAscope data (data includes mean of 4 control and 4 *Gdf11*^*cKO*^ brains). **G** Quantitative PCR analysis in isolated hippocampi from *Gdf11*^*flox/flox*^ and *Gdf11*^*cKO*^ mice. Analysis of TGFβ family members *Mstn*, *Tgfβ1*, *Tgfβ2*, *Tgfβ3* expression and GDF11 receptors *Alk4* and *Alk5* expression. Relative mRNA levels for all samples were normalized to the *Gdf11*^*flox/flox*^ group and error bars depict SEM. No statistically significant difference between groups was observed for any GDF11 family member or receptor studied. **H** Body weight (grams) measured for male and female *Gdf11*^*flox/flox*^ or *Gdf11*^*cKO*^ mice over varying lengths of time post 3 consecutive days of single tamoxifen IP injections (0.2 mg/gram b.w.). All statistics were calculated using two-tailed t-test. *p < 0.05, **p < 0.01

Proper recombination of the *Gdf11* allele in *Gdf11*^*cKO*^ mice was confirmed at the DNA level by genomic PCR (Fig. 3A, B). The efficiency of *Gdf11* depletion was assessed using quantitative PCR (qPCR) on brain tissue and select peripheral tissues from *Gdf11*^*cKO*^ mice and littermate *Gdf11*^flox/flox^ controls (Fig. 3C). *Gdf11* levels were normalized to the housekeeping gene *Hprt* and then normalized to the *Gdf11*^flox/flox^ group average. *Gdf11* levels were significantly reduced in *Gdf11*^*cKO*^ mice with an average percent reduction, reported by tissue type, of: whole brain (73%), hippocampus (79%), cerebellum (87%), kidney (80%), heart (72%), spleen (44%), lung (92%), muscle (97%), and liver (70%). All statistical analyses were performed using a two-tailed Student’s *t*-test and p < 0.05 for each tissue tested (Fig. 3C). We also quantified GDF11 protein levels using a direct LC–MS/MS assay on serum collected from *Gdf11*^*cKO*^ and *Gdf11*^*flox/flox*^ mice. We detected a statistically significant reduction of serum GDF11 in *Gdf11*^*cKO*^ mice relative to controls (16%, n = 9 per genotype, p < 0.05) (Fig. 3D). This relatively small reduction of GDF11 in serum is likely due, at least in part, to the low efficiency of *Gdf11* depletion in the spleen, a major source of circulating GDF11.

In order to confirm that *Gdf11* was depleted in the dentate gyrus, we performed quantitative RNAscope in-situ hybridization and observed a statistically significant 80% depletion of *Gdf11* in *Gdf11*^*cKO*^ mice relative to controls (Fig. 3E, F). This measurement of *Gdf11* depletion by RNAscope was consistent with qPCR results obtained using the same set of mice (Additional file 1: Fig. 4). We also tested whether depletion of *Gdf11* led to any compensatory changes in the expression of other TGFβ family members or GDF11 receptors. We investigated this possibility by performing qPCR on isolated hippocampal tissue from *Gdf11*^*cKO*^ and *Gdf11*^*flox/flox*^ mice. We observed no statistically significant change in the expression of *Mstn*, *Tgfβ1*, *Tgfβ2*, or *Tgfβ3* nor any change in the expression of GDF11 receptors *Acvr1b (Alk4)* or *Tgfβr1 (Alk5)* in the *Gdf11*^*cKO*^ mice (Fig. 3G). We also measured total body weight and observed no statistically significant difference between *Gdf11*^*cKO*^ and *Gdf11*^*flox/flox*^ mice in the 6 weeks post tamoxifen treatment (Fig. 3H).

### *Gdf11* depletion increases overall proliferation, the number of amplifying progenitors and the number of neuroblasts in the adult hippocampus

**Fig. 4:**
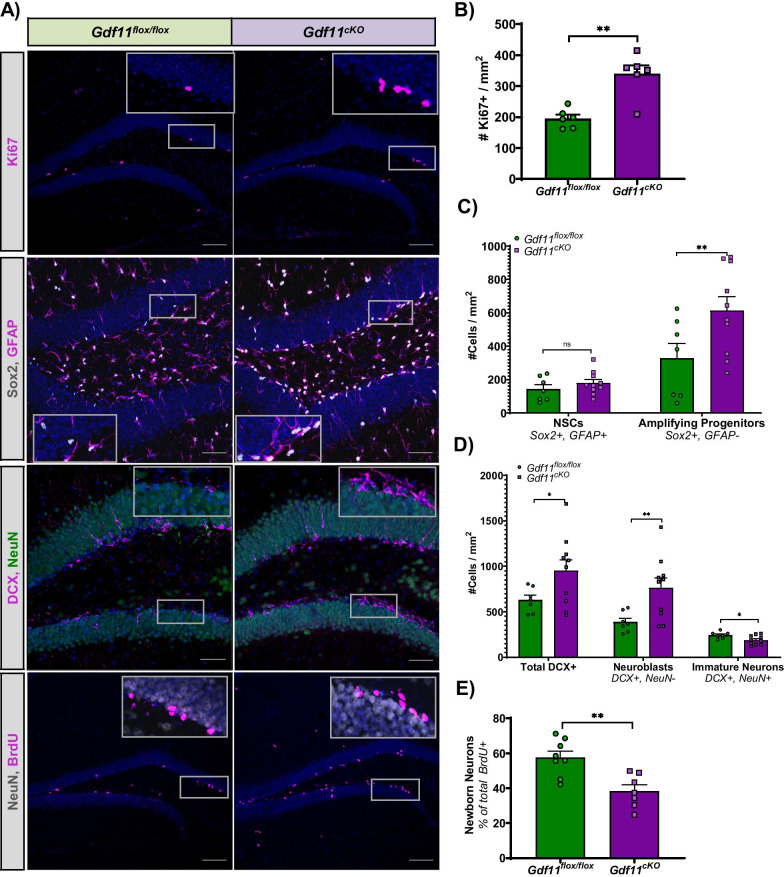
*Gdf11* depletion increases overall proliferation, increases the number of neural progenitors, and decreases the number of newborn neurons in the adult hippocampus. **A** Representative immunohistochemical micrographs comparing the expression of Ki67, Sox2/GFAP, DCX/NeuN, and NeuN/BrdU in the DG of *Gdf11*^*flox/flox*^ and *Gdf11*^*cKO*^ mice. Scale bar = 20 μm. **B** Quantification of overall proliferation as measured by the number of Ki67^+^ cells per DG mm^2^ (74% increase in *Gdf11*^*cKO*^ mice, n = 6 mice per group). **C** Quantification of the number of neural stem cells (25% nonsignificant increase in *Gdf11*^*cKO*^ mice, n = 7–10 mice per group) and the number of amplifying progenitors (87% increase, n = 7–10 per group). **D** Quantification of neuroblasts (DCX^+^, NeuN^−^) and immature neurons (DCX^+^, NeuN^+^), as measured by the proportion of DCX^+^ cells that colabeled with or without NeuN^+^ cells per DG mm^2^ (% decrease in *Gdf11*^*cKO*^ mice, n = 7–8 mice per group). **E** Quantification of newborn neurons, as measured by the proportion of BrdU^+^ cells that colabeled with NeuN^+^ cells per DG mm^2^ (50% decrease in *Gdf11*^*cKO*^ mice, n = 7–8 mice per group). See methods for details on cell classification scheme used. All statistics were calculated using two-tailed t-test.*p < 0.05 and **p < 0.01

Adult neurogenesis in the subgranular zone (SGZ) involves a series of distinct developmental stages where the proliferation and differentiation of neural progenitors ultimately generate new dentate granule neurons over the course of approximately 30 days [12]. Radial neural stem cells give rise to rapidly proliferating progenitors which exit the cell cycle and mature into newborn neurons. To investigate how GDF11 regulates neurogenesis, we injected *Gdf11*^*cKO*^ and *Gdf11*^*flox/flox*^ mice with tamoxifen once daily for three consecutive days and collected their brains 42 days later. First, we examined whether there were changes in overall proliferation and observed a 74% increase in Ki67^+^ cell number in the DG of *Gdf11*^*cKO*^ mice (p < 0.01) (Fig. 4A, B). We next sought to identify which proliferating cell types were impacted. We observed no significant change in the number of neural stem cells (Sox2^+^, GFAP^+^) but did observe an 87% increase in the number of amplifying progenitors (Sox2^+^) (Fig. 4A, C). These results are consistent with GDF11 playing an influential role on the more proliferative cell types, like amplifying progenitors, rather than radial neural stem cells which are largely quiescent. *Gdf11* depletion also led to a 51% increase in the total number of DCX^+^ cells which include both proliferating neuroblasts and postmitotic immature neurons (Fig. 4A, D). We used the neuronal marker NeuN to distinguish neuroblasts (NeuN^−^, DCX^+^) from immature neurons (NeuN^+^, DCX^+^). Interestingly, in *Gdf11*^*cKO*^ mice, we observed a 97% increase in the number of neuroblasts but a 23% decrease in the number of immature neurons relative to *Gdf11*^*flox/flox*^ mice (Fig. 4D). This result suggests that *Gdf11* depletion promotes the proliferation of neural progenitors (amplifying progenitors and neuroblasts) at the potential expense of proper cell cycle exit and differentiation into neurons.

### *Gdf11* depletion decreases the number of newborn neurons in the adult hippocampus

To test the hypothesis that *Gdf11* depletion leads to fewer newborn neurons, we performed a pulse chase experiment using the thymidine analog bromo-deoxyuridine (BrdU). *Gdf11*^*cKO*^ and *Gdf11*^*flox/flox*^ mice were injected once daily for three consecutive days with tamoxifen and 24 days later injected once daily for three consecutive days with BrdU. Animals were sacrificed 21 days following the final BrdU injection at day 48 post-tamoxifen. Under these conditions, some percentage of proliferating neural progenitor cells initially labeled by BrdU at day 24 should have differentiated into neurons. Newborn neurons were classified as the percent of total BrdU^+^ cells which co-stained with NeuN (Fig. 4A). We observed 50% fewer newborn neurons in *Gdf11*^*cKO*^ mice relative to *Gdf11*^*flox/flox*^ mice (p < 0.01) (Fig. 4A, E). Fewer newborn neurons after *Gdf11* reduction suggests GDF11 acts on neuroblast differentiation and/or the survival of newborn neurons.

## Discussion

**Fig. 5:**
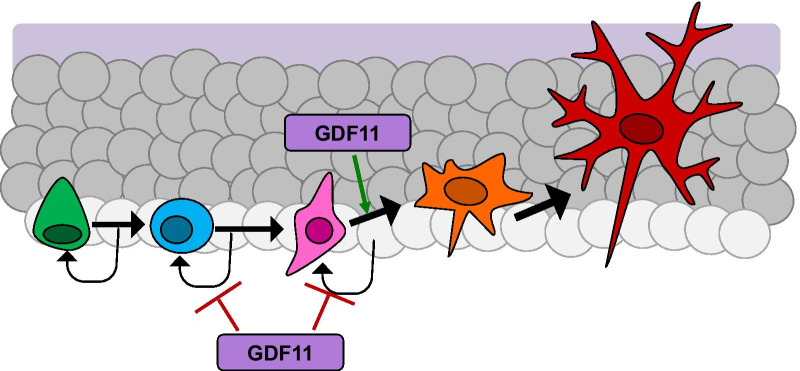
Model summarizing the role of GDF11 in adult hippocampal neurogenesis. GDF11 inhibits the proliferation of amplifying progenitors and neuroblasts under normal conditions. In *Gdf11*^*cKO*^ mice there is an increased number of these neural precursor cells. GDF11 promotes the differentiation of neuroblasts into immature neurons and in *Gdf11*^*cKO*^ mice there are fewer newborn neurons produced. Legend: Green (stem cell), blue (amplifying progenitor), pink (neuroblast), orange (immature neuron), red (mature neuron)

Until now, our understanding of GDF11’s expression and function in the CNS was largely limited to early developmental stages. Further, although *Gdf11* expression had previously been reported in the hippocampus of 2-week-old mouse pups, no prior study had explored the function of GDF11 in this brain region [2]. Our experiments provide the first detailed, quantitative histological characterization of the cellular sources for *Gdf11* in the adult mouse brain. We show *Gdf11* does continue to be expressed throughout the hippocampus, both during young adulthood and with advanced age. Our RNAscope findings are consistent with results from a recent qualitative study of GDF11 protein expression in young adult rat brains [13]. In the hippocampal neurogenic niche, we detected *Gdf11* expression by both neural progenitors and mature neurons. This was based on the coexpression of *Gdf11* mRNA puncta with cell type specific markers in a cell dense region and as a result expression of *Gdf11* by additional nonneuronal cells cannot be ruled out. Using *Gdf11*^*cKO*^ mice, we demonstrate for the first time that GDF11 is required for the normal proliferation of adult neural progenitor cells. We reached this conclusion after observing, in the absence of *Gdf11*, an increase in overall Ki67^+^ cells which coincided with an increase in the number of both Sox2^+^ amplifying progenitors and DCX^+^ neuroblasts. Despite an accumulation of DCX^+^, NeuN^−^ neuroblasts in *Gdf11*^*cKO*^ mice, we observed a reduction in NeuN^+^ newborn neurons. This was indicated by a decrease in the number of immature neurons 6 weeks after *Gdf11* depletion and by a decline in newly generated neurons which incorporated BrdU. This decline in BrdU^+^, NeuN^+^ newborn neurons was observed despite an increase in the overall number of BrdU^+^ cells in *Gdf11*^*cKO*^ mice, further supporting the possibility these BrdU^+^, NeuN^−^ cells represent neural progenitors that cannot effectively differentiate. Altogether, our results underscore how endogenous GDF11 suppresses hippocampal neurogenesis during development and continuing through adulthood (Fig. 5). This reinforces the notion that systemic rGDF11, which increases adult hippocampal neurogenesis, is likely to act without crossing the BBB, consistent with prior work from our laboratory suggesting that it signals via brain vasculature [8]. This is interesting in that it further suggests that the same molecule can have opposite effects on an essential CNS process, depending on *where* and on which cells it acts. A lack of change in *Gdf11* with advanced age may suggest that other regulatory factors which do change with age are responsible for age-related declines in neurogenesis, including the possibility of an age-dependent change in a GDF11 regulator. It is also possible that the effect of GDF11 on neurogenesis changes between development and young adult mice, when it has the same inhibitory effect, and old mice, when it hypothetically would have a stimulatory effect. Even if this were true, it would not explain how GDF11, whose levels do not change, could account for the decline in neurogenesis.

### Potential mechanism underlying GDF11 inhibition of NPC proliferation and promotion of newborn neuron number in the adult hippocampus

Upon *Gdf11* depletion, we observed an increase in NPC proliferation that coincided with a decrease in newborn neurons. This decrease in newborn neurons could be the result of impaired differentiation of neural progenitors, impaired newborn neuron survival, or a combination of both mechanisms. Support for a direct mechanism involving impaired differentiation comes from GDF11 developmental studies. In the embryonic olfactory epithelium and spinal cord, GDF11 is produced by mature neurons and negatively regulates proliferation of nearby neural progenitors by promoting their cell cycle exit. This is achieved in part through the transcriptional activation of the cyclin-dependent kinase inhibitor p27^kip1^, which arrests progenitor cells in the G_1_ phase of the cell cycle [3, 7]. NPCs must exit the cell cycle in order to differentiate, and excessive proliferation caused by embryonic *Gdf11* deletion is responsible for the decreased ability of NPCs to differentiate into mature neuronal subtypes [5, 7]. Interestingly, p27^kip1^ is expressed in the adult hippocampus by neural progenitor cells and immature neurons [14, 15]. Loss of p27^kip1^ leads to an increase in neural progenitor proliferation and a decrease in the number of newborn neurons in the adult dentate gyrus [14, 15]. In vitro assays utilizing adult hippocampal neural stem cells further demonstrate that the mRNA levels of *Cdkn1b*, which encodes p27^kip1^ protein, increases during neuronal differentiation and positively correlated with markers of neuronal maturity [14, 16]. Given the ability of GDF11 to regulate p27^kip1^ expression during development, we plan to investigate whether a similar mechanism may underlie GDF11 functions during adult hippocampal neurogenesis. A direct regulatory effect of GDF11 on differentiation is also consistent with our data demonstrating that treatment of neurospheres derived from adult mouse brain with recombinant GDF11 decreases their proliferation [8].

Experiments investigating effects of other TGF-β family members are also consistent with GDF11 being neuroprotective for immature neurons in the hippocampus. GDF11 preferentially signals through the Alk5 receptor, and overexpression of a constitutively active form of *Alk5* in the adult hippocampus promotes the survival of newborn neurons [17]. Conversely, conditional deletion of *Alk5* from postmitotic immature and mature neurons results in fewer newborn neurons [17]. In addition, TGF-β1, which also binds Alk5, has been linked to hippocampal neuron survival. For example, pretreatment with TGF-β1 protects cultured rat hippocampal neurons from apoptotic cell death, NMDA-mediated excitotoxicity, oxidative stress, and glutamate neurotoxicity [18–21]. Finally, astroglial overexpression of *Tgf-β1* in adult mice protects against neurodegeneration in acute, excitotoxic, and chronic injury models [22]. However, it may be important to note that the main producers of TGF-β1 in the adult hippocampus are glia and immune cells, while our data demonstrate that GDF11 is primarily produced by neuronal and oligodendrocyte-lineage cells [23, 24]. Understanding how GDF11 may act independently or in concert with TGF-β1 to regulate hippocampal neuron survival and the implications of their different cellular sources would be an interesting area for future study. Our data are consistent with the view, established in studies investigating developing tissue, that neuronal GDF11 acts as an inhibitor of NPC proliferation. However, GDF11 in the adult brain is also expressed in non-neuronal cells. Thus, future investigations into the extent to which these non-neuronal sources contribute to changes in NPC proliferation and/or neuron survival could also yield new insights.

### Role for GDF11 in non-neurogenic brain regions

We found that GDF11 is relatively highly expressed in non-neurogenic regions like the thalamus, habenula, and cerebellum. Interestingly, we observed the highest density of *Gdf11* expression in the habenula and our results align with a recent habenula focused single-cell RNA-sequencing paper which reported *Gdf11* within the top 25% of highly expressed genes in the region [25]. The habenula contains primarily cholinergic neurons and has been shown to play an important role in cognition dependent executive functions and inhibitory control [26]. We also detected strong expression of *Gdf11* in the cerebellum, most likely in Purkinje cells in the molecular layer [27]. This result is consistent with data from a single-cell sequencing study which specifically profiled cells isolated from the adult mouse cerebellum [28]. Again, this is consistent with known in vitro effects of GDF11 on neurite outgrowth, dendrite formation, and synapse formation [29–31].

### Considerations for GDF11-based CNS therapies

Dysfunctional adult hippocampal neurogenesis has been linked to several CNS disorders including age-related declines in cognitive function, stroke, Alzheimer’s disease, and epilepsy [32–36]. As a result, identifying therapeutic candidates that target neurogenesis is an area of robust research. Our lab previously demonstrated that systemic injection of rGDF11 can promote neurogenesis in aged mice without crossing the blood brain barrier [8, 9]. This suggests that GDF11 signaling is highly location and cell type dependent, which underscores the significance of considering route of administration for any rGDF11 based therapy. When the goal is to promote neurogenesis, our data indicate rGDF11 treatment should avoid direct contact of rGDF11 with NPCs. This is particularly relevant in the context of stroke, where the blood brain barrier is known to break down transiently, and rGDF11 treatment has been proposed as a means to improve functional recovery by promoting neurogenesis [37–39]. Under those conditions, however, systemically administered GDF11 might improve revascularization and, possibly, increase neuronal survival, processes that would be particularly important outside of the of relatively restricted neurogenic zones. However, in some diseases, like epilepsy, aberrant increases in hippocampal neurogenesis can exacerbate symptoms, and administration of neurogenesis inhibitory factors is then desirable [36, 40]. In this context, upregulating locally expressed GDF11 could potentially mitigate the negative consequences of seizure-induced neurogenesis. Given the negative regulatory role of GDF11 in neurogenesis, altering endogenous GDF11 expression locally in the neurogenic niche could be an alternative therapeutic strategy or even worth pairing with systemically administered rGDF11 targeting vascular cells depending on the treatment goal.

## Conclusion

Our study provides new insight into the distribution of GDF11 in the adult mouse brain and presents evidence that GDF11 continues to play a similar role in neurogenesis during adulthood as it does during development. We show how GDF11 deficiency causes an increase in NPC proliferation and a decrease in the number of newborn hippocampal neurons. These data support an indirect mechanism of action for how *systemically administered* rGDF11 promotes neurogenesis, as direct contact with rGDF11 would presumably inhibit NPC proliferation. The microenvironment of the hippocampal neurogenic niche is highly regulated to enable a continuous source of cells for growth, maintenance, and repair. Identification of factors like GDF11 which inhibit neurogenesis but support mature neurons can help facilitate the development of strategies to promote stem cell regeneration and maturation.

## Methods

### Mice

Animal care and experimental procedures were approved by the Animal Care and Use Committee of Harvard University (AEP no. 10-23) and are in compliance with state and federal laws. All mice were housed in the Harvard Biolabs Animal Facility under standard conditions Both male and female C57BL/6J mice (JAX no. 000664) were used at 2–3 months of age for characterization of *Gdf11* expression. Old mice used were 18 months of age.

To generate our tamoxifen-inducible *Gdf11* knockout model, mice containing a floxed *Gdf11* allele with loxP sites flanking exons 2 and 3 of *Gdf11*[1] were bred with Tg(cre/Esr1)5Amc/J mice, obtained from Jackson labs (JAX no. 004682) [11]. All mice were on a C57Bl/6J background. Genotyping was performed using primers that distinguish *Gdf11* wt, flox, ∆ alleles and Cre^ER^+ or Cre^ER^− status. Primers used are summarized in Additional file 2: Table 1. We used age-matched male and female KO and control mice for all analyses.

### Tamoxifen preparation and administration

To activate the latent Cre^ER^ recombinase, *Gdf11*^*cKO*^ and control mice were administered tamoxifen (TAM) via intraperitoneal (IP) injections once daily for three consecutive days starting at 2–3 months of age. TAM was dissolved in a corn oil (Sigma)/10% ethanol solution and injected at a concentration of 0.02 mg/gram body weight. For all litters, Cre-ER negative and Cre-ER positive mice were mated in order to generate littermate control within each experiment.

### 5-Bromo-2′-deoxyuridine injections

5-Bromo-2′-deoxyuridine (BrdU) (Sigma-Aldrich, St. Louis) was dissolved in sterile PBS at concentration of 10 mg/mL and prepared fresh for each daily use. An intraperitoneal injection of BrdU (100 mg/kg body weight) was administered once daily for three consecutive days starting at day 24 following the final injection of tamoxifen.

### RNA isolation, cDNA synthesis, and quantitative PCR

For all qPCR experiments, tissue was isolated and snap frozen in liquid nitrogen. Tissues were homogenized in Trizol (Thermo Fisher) and the aqueous phase was collected according to the manufacturer’s protocol. The aqueous phase was mixed with equal volume of 70% ethanol, purified using the RNeasy mini kit (Qiagen), and final RNA concentration was calculated using NanoDrop (Thermo Fisher). RNA samples were reverse transcribed into cDNA with the iScript cDNA synthesis kit (Bio-Rad no. 170-8891) following the manufacturer’s instructions. The resulting cDNA was then processed for qPCR analysis with predesigned primers and the Fast SYBR Green Master Mix (Life Technologies no.4385614) in a QuantStudio 12 K Flex Real-Time PCR System (Applied Biosystems). PCR primer sequences are listed in Additional file 2: Table 1. Before data analysis, we examined the melting curves for each reaction and included only those with a single peak at the expected melting temperature. The fold change in gene expression was determined by the 2^−DDCT^ method [41]**,** and all values were normalized to the endogenous expression of *Hprt*; a housekeeping gene. Each sample was repeated in technical triplicates.

### RNAscope in situ hybridization

#### Tissue processing

For sample preparation, mice were CO2 anesthetized, and brains were rapidly extracted and embedded in OCT (Tissue Tek) on dry ice, and then stored at − 80 °C until further processing. We collected 14-μm cryostat sections and RNAscope hybridizations were carried out according to the manufacturer’s instructions, using the RNAscope Multiplex Fluorescent Manual Assay kit (Advanced Cell Diagnostics, #320850). Briefly, thawed sections were dehydrated in sequential incubations with ethanol, followed by 30-min Protease IV treatment and washing in 1 × PBS. Appropriate combinations of hybridization probes were incubated for 2 h at 40 °C, followed by four amplification steps, 4,6-diamidino-2-phenylindole (DAPI) counterstaining, and mounting with Prolong Gold mounting medium (Thermo Fisher Scientific no. P36930). For single-probe analysis, probes were labeled with the fluorophore Atto-550 nm. For each mouse, 3–4 bregma-matched sections were imaged. Images were acquired with a Zeiss LSM 880 Confocal Microscope with identical settings across all samples and represented as maximum intensity projections of acquired confocal z stacks. For coexpression analysis, cell-type specific probes were labeled by fluorophore Alexa-488 nm or Atto-647 nm, while target probes were labeled by fluorophore Atto-550 nm. An empty channel was collected for every image processed to account for any potential autofluorescence. For a complete summary of each RNAscope probe tested see Additional file 2: Table 2.

#### Quantification

The most current version of CellProfiler at the time (v.3) was downloaded and used for quantification with a custom image analysis pipeline [42]*.* Only puncta with a diameter between 4 and 8 pixels that were located within the perinuclear space (defined as within 70 pixels of the DAPI-positive nuclei) were quantified. To minimize the quantification of background puncta, puncta detection settings were optimized with RNAscope micrographs collected using a negative control probe (DapB, dihydrodipicolinate reductase, a *Bacillus subtilis* bacterial gene). To address potential concerns surrounding cross-detection of *Gdf11* with its highly homologous family member *Mstn*, RNAscope was performed comparing *Gdf11* probe and *Mstn* probes (Additional file 1: Fig. 2). A technical limitation to note is the use of a standardized distance measure for defining the perinuclear space around DAPI positive nuclei and different segmentation techniques may result in slightly different assignment of puncta per cell.

### Immunohistochemistry

#### Tissue processing

For preparation of free-floating tissue sections, mice were sacrificed at indicated time points and perfused transcardially with 4% paraformaldehyde (PFA) in PBS under deep anesthesia. Brains were removed and post-fixed overnight in 4% PFA. Brains were then transferred to 30% sucrose for 24 h before being embedded in OCT (Tissue Tek) on dry ice. Each brain was cut using a Leica CM1950 cryostat and immunostaining was performed using 30-μm free-floating coronal sections. Sections were permeabilized and blocked in 10% normal goat or donkey serum and 0.1% Triton X-100 in PBS for 1 h at room temperature. For BrdU immunostaining, sections were pre-treated with 2 M HCL for 30 min at 37C prior to permeabilization and blocking steps. Sections were then incubated overnight at 4C with primary antibodies. Primary antibodies and dilutions used for IHC are summarized in Additional file 2: Table 3. Alexa Flour 488, 568, 647 secondary antibodies were used at 1:500 dilution in 1% normal goat or donkey serum in PBS for 1 h at room temperature. DAPI (4′,6-Diamidino-2-Phenylindole, Dilactate) was used to label nuclei (Life Technologies #D3571). Coverslip mounting was performed using Dako Fluorescence Mounting Medium (Agilent #S302380-2).

#### Quantification

The most current version of QuPath at the time, v0.2.02, was downloaded from the QuPath homepage located at Github (https://QuPath.github.io/). QuPath allows the user to determine how much RAM it may access at once; we allowed it 6 GB and all analysis was done under this constraint. The 2-part Bio-Formats extension necessary to open Zeiss confocal image filetypes were downloaded and installed. Part 1 was from the Bio-Formats homepage (http://www.openmicroscopy.org/bio-formats/downloads/) and Part 2, specific to QuPath, was downloaded from the QuPath Github page (https://github.com/qupath/qupath-bioformats-extension).

We created a new “Project” within QuPath for each stain analyzed, in order to allow for quantification of the entire project automatically with the appropriate code, rather than re-executing the steps for each image. All projects included a tissue area detection step where we manually traced the region of interest labeled by DAPI. All images analyzed were maximum intensity projections of z-stacks acquired on a Zeiss LSM 880 confocal microscope. Sections analyzed were always bregma matched between the Flox control and GDF11 KO mice and 2–3 technical replicates were included per mouse per stain. Quantification parameters for each stain analyzed are as follows:

- Proliferation: the number of Ki67^+^ cells per granular cell layer (GCL) were counted in a 1-in-18 series of sections (540 um apart) throughout the hippocampus and immunopositive cells were summed across sections and expressed as number per mm^2^.
- Neural stem and amplifying progenitor cells: the total number of Sox2^+^ cells per GCL were counted in bregma matched sections. Neural stem cells were classified as Sox2^+^ cells which co-expressed GFAP and exhibited radial morphology. Amplifying progenitor cells were classified as Sox2^+^ cells which failed to meet the definition of neural stem cells. Quantification of both cell types identified were expressed as number of cells per mm^2^.
- Neuroblasts and immature neurons: the total number of DCX^+^ cells per GCL were counted in bregma matched sections. Neuroblasts were classified as DCX^+^ cells which did not express the neuronal marker NeuN. Immature neurons were classified as DCX^+^ cells which co-expressed NeuN. Quantification of both cell types identified were expressed as number of cells per mm^2^.
- Newborn neurons: the number of BrdU^+^ cells per GCL were counted in a 1-in-18 series of Sects. (540 um apart) throughout the hippocampus and immunopositive cells were summed across bregma matched sections and expressed as number per mm^2^. Newborn neurons were classified as BrdU^+^ cells which co-expressed the neuronal marker NeuN.

### Imaging equipment and settings

Images were acquired using Zeiss LSM 880 Confocal Microscope with either ×10, ×20, or ×40 magnification. Lasers used include the following: Alexa Flour 488, Atto 550, and Atto 647.

### Serum collection and LC–MS/MS analysis

Mice were anesthetized, decapitated, and trunk blood was transferred to serum separator tubes (VWR #VT365967). Tubes were spun at 2000 g for 5 min, and serum was transferred to clean low-binding microcentrifuge tubes and stored at − 80 °C until further processing. Samples (100 μL) were submitted to the Brigham Research Assay Core at Brigham and Women’s Hospital and liquid chromatography tandem mass spectrometry (LC–MS/MS) was performed as described previously[43].

### Statistical analysis

All statistical analyses were performed using the GraphPad Prism software. Results were expressed as mean ± standard error unless otherwise specified. Comparisons between groups were made by two-tailed Student’s *t*-test when appropriate. Statistical significance was designated as p < 0.05 or p < 0.01 within each figure shown.

## Supplementary Information

**Additional file 1: Figure 1.***Gdf11* coexpression data in nonneuronal cells of the choroid plexus and hippocampal dentate gyrus. Representative RNAscope micrographs from a 3-month-old male C57BL/6 mouse probed for *Gdf11* (white), probed for a cell specific marker (pink or green), and stained with DAPI (blue). A) Cell types probed include: SVZ neuroblasts (*Dcx*), endothelial cells (*Pecam1*), and choroid plexus epithelial cells (*Ttr*). B) Cell types probed include: oligodendrocytes (*Olig1*), oligodendrocyte precursor cells (OPCs) (*Pdgfra*), microglia (*Itgam*), and endothelial cells (*Pecam1*). Gray boxes mark the regions chosen for the enlarged inserts below each micrograph shown. Scale bar = 20 μm. **Figure 2.** *Gdf11* is expressed at higher levels than *Mstn* in young adult dentate gyrus. A) Representative RNAscope micrographs from a 3-month-old male C57BL/6 mouse probed for *Gdf11* (white, left panel) or *Mstn* (white, right panel). B) Bar graph depicting the quantification of RNAscope data (n = 4 mice, error bars represent SEM). **Figure 3.** *Gdf11* expression in the hippocampus does not change with age. A) Representative RNAscope micrographs from either a young (3 month old) or old (18 month old) male C57BL/6 mouse probed for *Gdf11* (white puncta) and stained with DAPI (blue). Scale bar = 20 μm. B) Quantification of RNAscope data (mean of 4 mice per age studied, error bars depict SEM, no within region comparisons were statistically significant). C) Quantitative PCR analysis of *Gdf11* expression in hippocampus tissue isolated from young and old male C57BL/6 mice (n = 3 young hippocampi vs n = 4 old hippocampi). *Hprt* was used as a housekeeping gene. Relative mRNA levels for all samples are normalized to the young mice age group and error bars depict SEM. Statistics were calculated using two-tailed t-test. **Figure 4.** Comparing quantification of *Gdf11* by RNAscope and qPCR in *Gdf11*^*cKO*^ mice vs controls. A) Scatterplot showing the quantification of RNAscope performed on mouse hippocampi (dentate gyrus) probed for Gdf11.N = 4 GDF11^cKO^ brains and n = 4 controls (2 *Gdf11*^flox/flox^ and 2 *CAG-CreER;GDF11*^*flox/flox*^ mice). B) Quantitative PCR analysis of *Gdf11* expression in isolated hippocampi from *Gdf11*^*flox/flox*^, *Gdf11*^*cKO*^, and *CAG-Cre*^*ER*^*;* GDF11flox/flox mice. *Hprt* was used as a housekeeping gene. Relative mRNA levels for all samples are normalized to control mice, and error bars depict SEM. Statistics were calculated using a two-tailed t-test.

**Additional file 2: Table 1.** Primer Sequences. **Table 2.** RNAscope Probes. **Table 3.** Primary antibodies and dilutions.

## Data Availability

The datasets generated and analyzed during the current study are available from the corresponding author on reasonable request.

## Abbreviations

BBB: Blood–brain barrier
BrdU: Bromodeoxyuridine
CNS: Central Nervous System
DG: Dentate gyrus
GCL: Granular cell layer
GDF11: Growth Differentiation Factor 11
IP: Intraperitoneal
NSC: Neural stem cell
OPC: Oligodendrocyte precursor cell
rGDF11: Recombinant GDF11
SGZ: Subgranular zone
SVZ: Subventricular zone
TAM: Tamoxifen
TGF-β: Transforming growth factor-beta
