# Supplementary material for: GDF11 expressed in the adult brain negatively regulates hippocampal neurogenesis
Source: Mol Brain. 2021 Sep 6;14:134. doi: 10.1186/s13041-021-00845-z (PMC8422669; doi:10.1186/s13041-021-00845-z)
Supplement: Supplementary file 1 — Additional file 1: Figure 1.Gdf11 coexpression data in nonneuronal cells of the choroid plexus and hippocampal dentate gyrus. Representative RNAscope micrographs from a 3-month-old male C57BL/6 mouse probed for Gdf11 (white), probed for a cell specific marker (pink or green), and stained with DAPI (blue). A) Cell types probed include: SVZ neuroblasts (Dcx), endothelial cells (Pecam1), and choroid plexus epithelial cells (Ttr). B) Cell types probed include: oligodendrocytes (Olig1), oligodendrocyte precursor cells (OPCs) (Pdgfra), microglia (Itgam), and endothelial cells (Pecam1). Gray boxes mark the regions chosen for the enlarged inserts below each micrograph shown. Scale bar = 20 μm. Figure 2. Gdf11 is expressed at higher levels than Mstn in young adult dentate gyrus. A) Representative RNAscope micrographs from a 3-month-old male C57BL/6 mouse probed for Gdf11 (white, left panel) or Mstn (white, right panel). B) Bar graph depicting the quantification of RNAscope data (n = 4 mice, error bars represent SEM). Figure 3. Gdf11 expression in the hippocampus does not change with age. A) Representative RNAscope micrographs from either a young (3 month old) or old (18 month old) male C57BL/6 mouse probed for Gdf11 (white puncta) and stained with DAPI (blue). Scale bar = 20 μm. B) Quantification of RNAscope data (mean of 4 mice per age studied, error bars depict SEM, no within region comparisons were statistically significant). C) Quantitative PCR analysis of Gdf11 expression in hippocampus tissue isolated from young and old male C57BL/6 mice (n = 3 young hippocampi vs n = 4 old hippocampi). Hprt was used as a housekeeping gene. Relative mRNA levels for all samples are normalized to the young mice age group and error bars depict SEM. Statistics were calculated using two-tailed t-test. Figure 4. Comparing quantification of Gdf11 by RNAscope and qPCR in Gdf11cKO mice vs controls. A) Scatterplot showing the quantification of RNAscope performed on mouse hippocampi (dentate gyru [file 13041_2021_845_MOESM1_ESM.pdf]

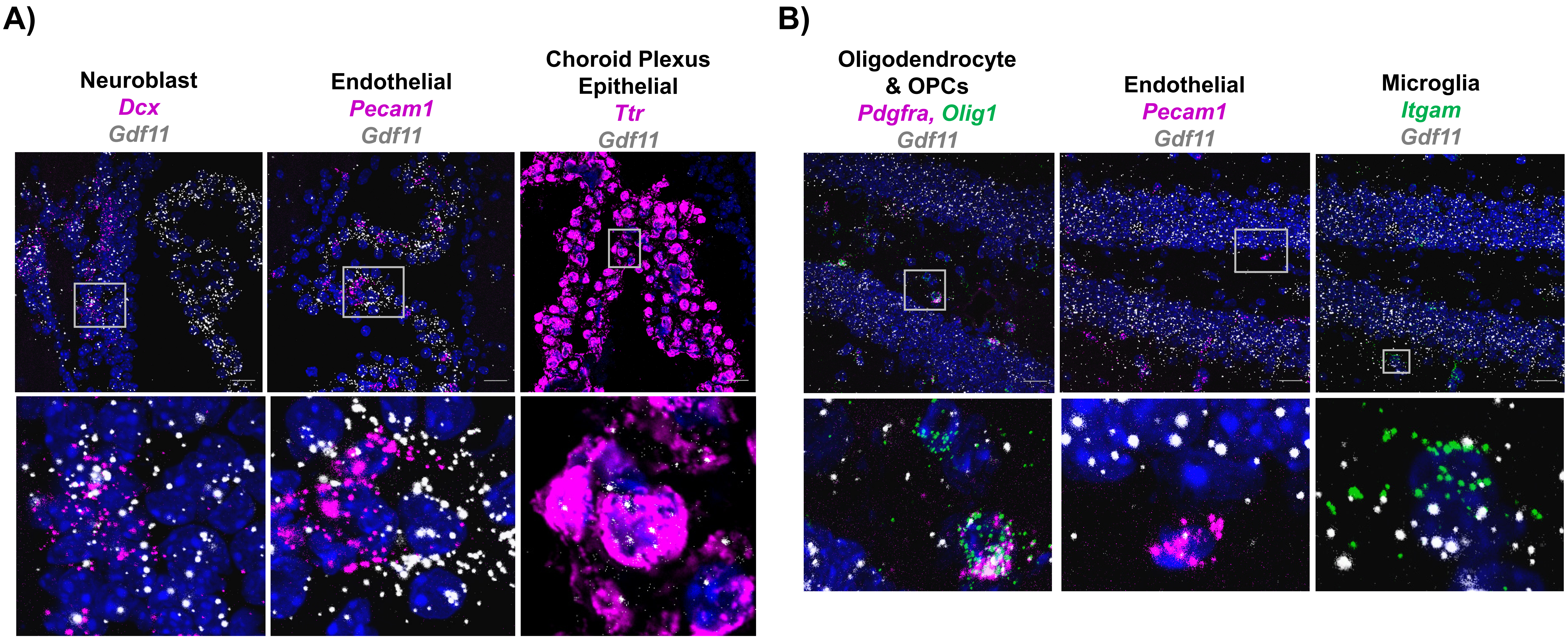

Additional Figure 1

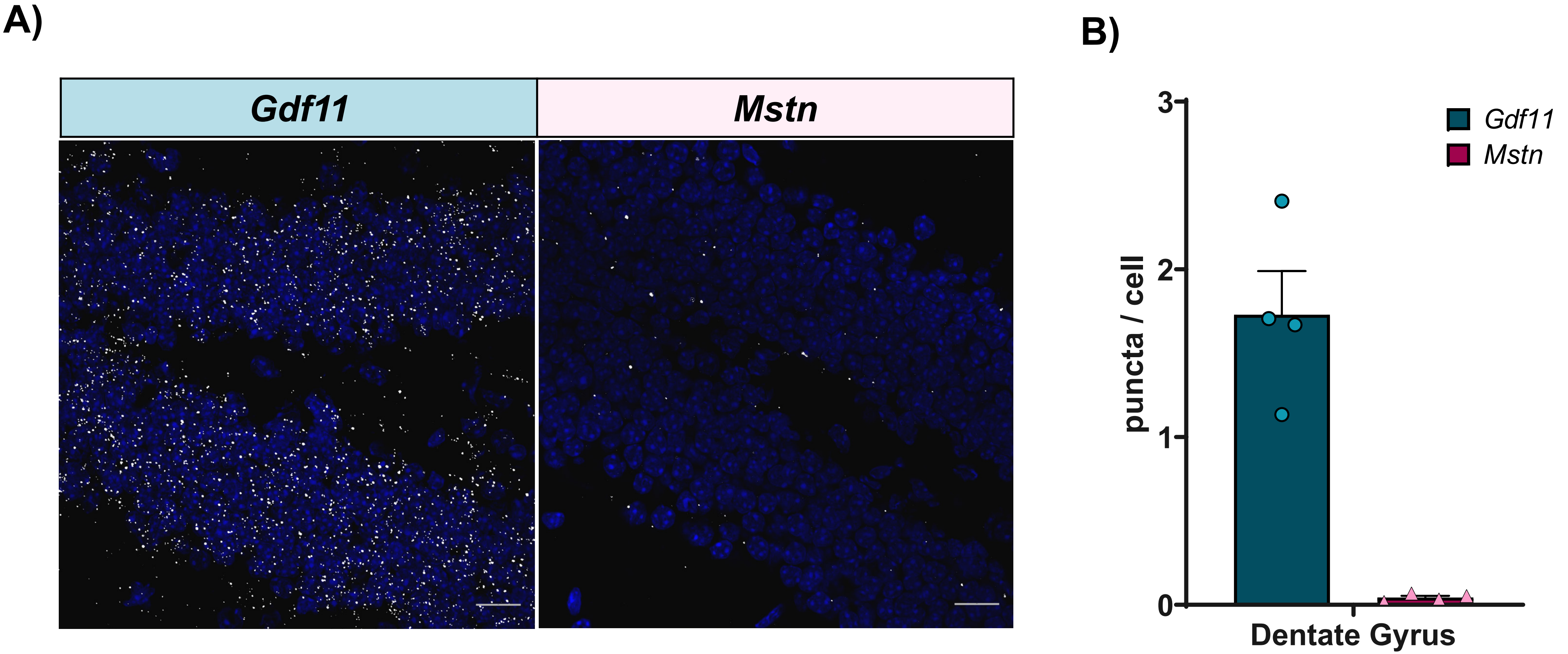

Additional Figure 2

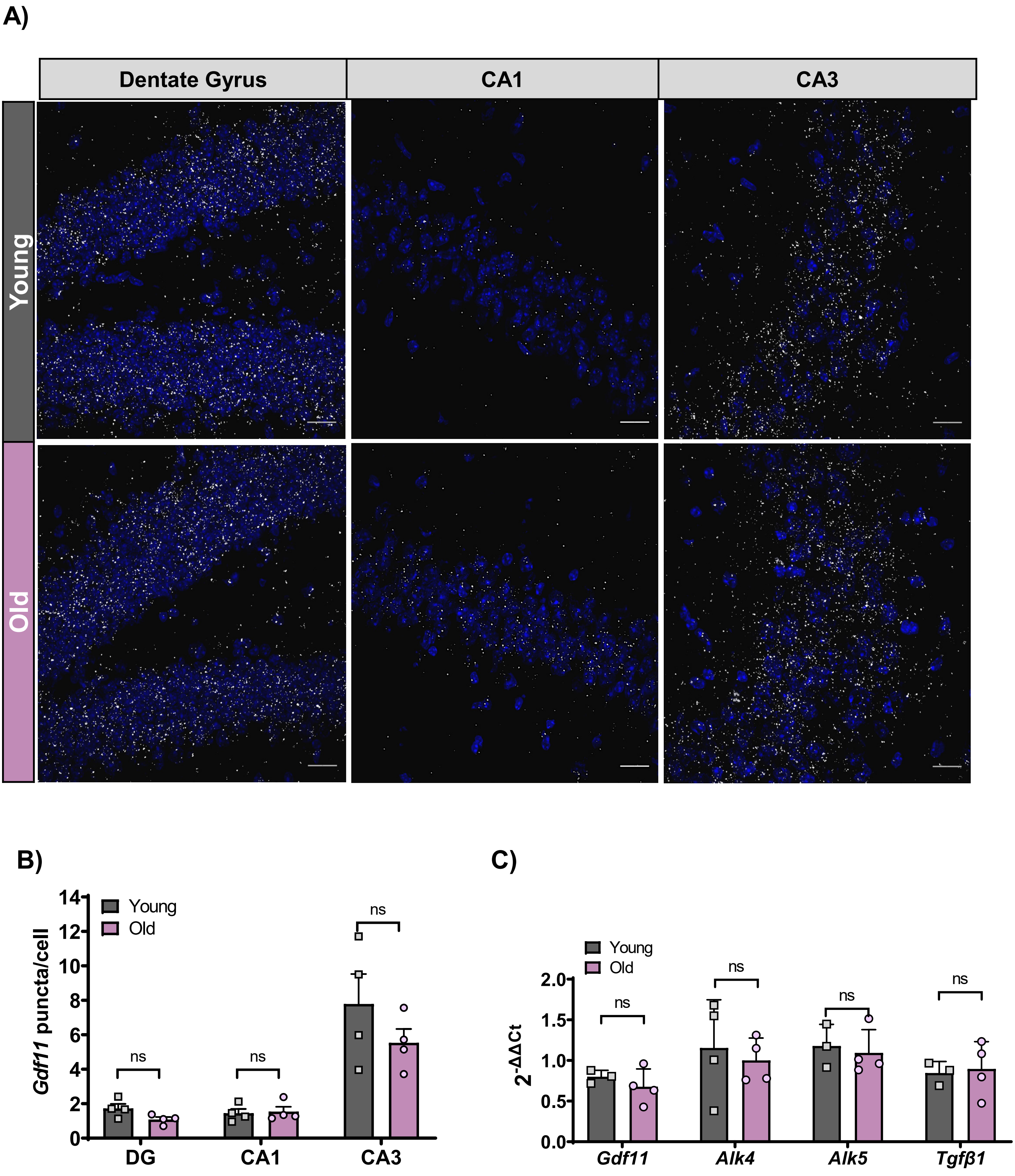

Additional Figure 3

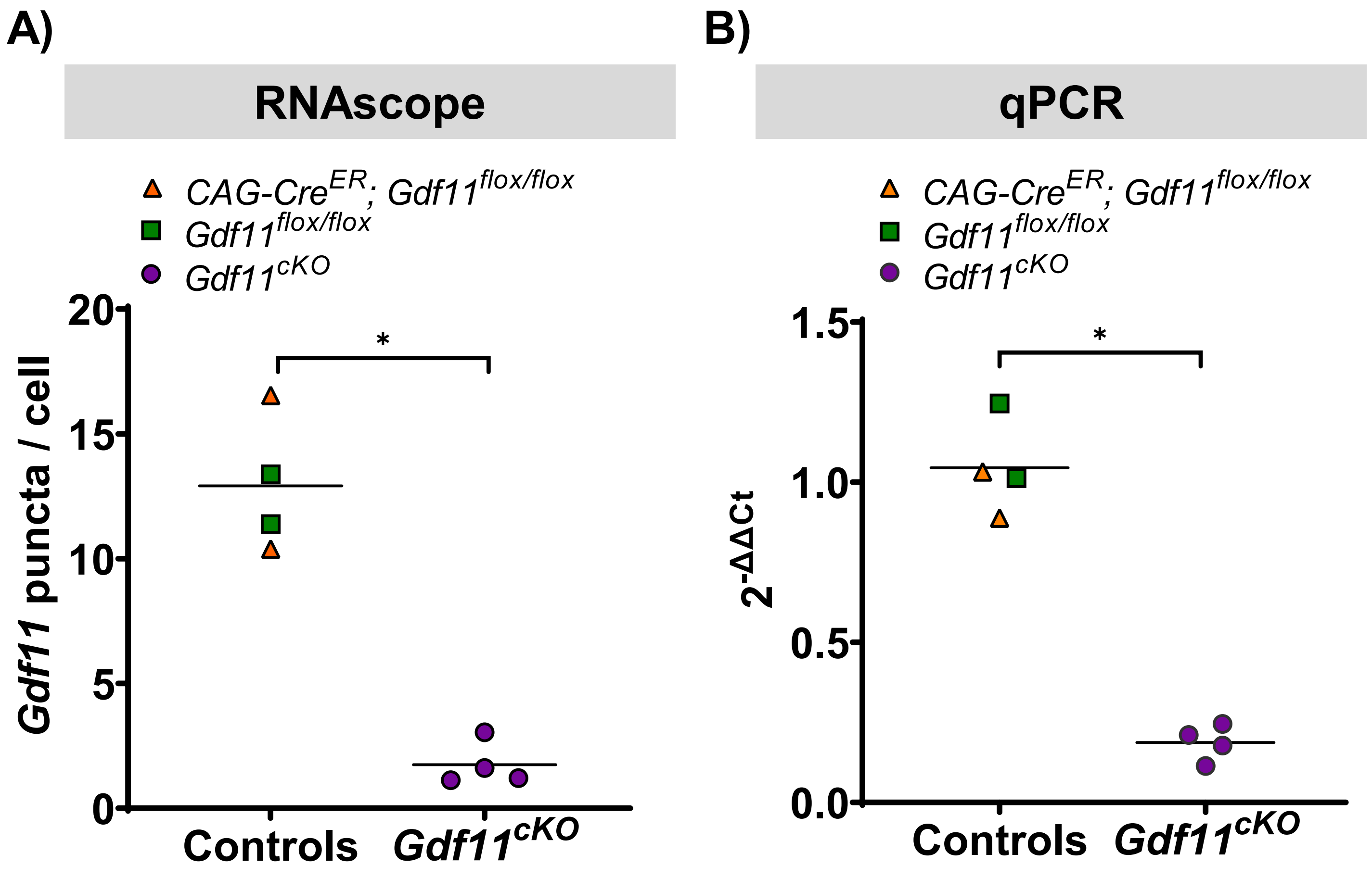

Additional Figure 4
