# Supplementary material for: GDF11 expressed in the adult brain negatively regulates hippocampal neurogenesis
Source: Mol Brain. 2021 Sep 6;14:134. doi: 10.1186/s13041-021-00845-z (PMC8422669; doi:10.1186/s13041-021-00845-z)
Supplement: Supplementary file 2 — Additional file 2: Table 1. Primer Sequences. Table 2. RNAscope Probes. Table 3. Primary antibodies and dilutions. [file 13041_2021_845_MOESM2_ESM.docx]

**Additional Tables**

### Additional Table 1. Primer Sequences

| **Primer Name** | **Sequence (5’-3’’) or Source** | **Purpose** |
| --- | --- | --- |
| *GDF11_a* | ATGCAGATGGTAATACTTGGG | PCR of GDF11 wt, flox, and ∆ alleles |
| *GDF11_b* | AAGGCTTGGGAAGCAGGCAAG | PCR of GDF11 wt, flox, and ∆ alleles |
| *GDF11_c* | AGGTATGGTTAGGGTGTGGAG | PCR of GDF11 wt, flox, and ∆ alleles |
| *CAG-Cre_F* | GCT AAC CAT GTT CAT GCC TTC | PCR for presence of Cre-ER driver |
| *CAG-Cre_R* | AGG CAA ATT TTG GTG TAC GG | PCR for presence of Cre-ER driver |
| *Hprt* | Qiagen # PPM03559F | Real-time PCR |
| *mMSTN ex2_F4* | TCTTGCTGTAACCTTCCCAGG | Real-time PCR |
| *mMSTN ex3_R4* | CAAAATCGACCGTGAGGGGG | Real-time PCR |
| *mTGFb1 ex2_F1* | TACGTCAGACATTCGGGAAGC | Real-time PCR |
| *mTGFb1 ex3_R1* | GACAGCCACTCAGGCGTATC | Real-time PCR |
| *mTGFb2 ex1_F1* | TACTGCAGGAGAAGGCAAGC | Real-time PCR |
| *mTGFb2 ex2_R1* | CTCTGGCTTTGGGGTTTTGC | Real-time PCR |
| *mTGFb3 ex3_F1* | CTTCGACCGGATGAGCACATAG | Real-time PCR |
| *mTGFb3 ex4_R1* | TGCTGATTTCCAGACCCAAGT | Real-time PCR |
| *mTGFBR1 ex3_F1* | CACCGTGTGCCAAATGAAGA | Real-time PCR |
| *mTGFBR1 ex4_R1* | GCCAAACTTCTCCAAACCGAC | Real-time PCR |
| *mAlk4 ex3_F1* | GTCTACGACCTCTCCACGTC | Real-time PCR |
| *mAlk4 ex4_R1* | ACGGTCTGGTAGATCTCTGCT | Real-time PCR |

### Additional Table 2. RNAscope Probes

| **Probe Name** | **ACD Product No.** | **Ch.** | **Target** |
| --- | --- | --- | --- |
| *Gdf11* | 425221-C1 | 1 | Growth Differentiation Factor 11 |
| *Mstn* | 402801-C1 | 1 | Myostatin |
| *Dcx* | 478671-C2 | 2 | Cell-type marker (Neuroblasts) |
| *Map2* | 431151-C3 | 3 | Cell-type marker (Mature Neurons) |
| Negative Control | 320871 | - | DapB (Bacillus subtilis strain) |

### Additional Table 3. Primary antibodies and dilutions

| **Antibody** | **Host** | **Dilution** | **Company** | **Product #** |
| --- | --- | --- | --- | --- |
| BrdU | Rat | 1:200 | Abcam | ab6326 |
| DCX | Rabbit | 1:200 | Abcam | ab18723 |
| GFAP | Chicken | 1:1000 | Abcam | ab4674 |
| Ki67 | Rabbit | 1:200 | Abcam | ab15580 |
| NeuN | Mouse | 1:500 | Millipore | MAB377 |
| Sox2 | Rabbit | 1:200 | Abcam | ab97959 |
